# Genomic Island Prediction via Chi-Square Test and Random Forest Algorithm

**DOI:** 10.1155/2021/9969751

**Published:** 2021-05-24

**Authors:** Mbulayi Onesime, Zhenyu Yang, Qi Dai

**Affiliations:** College of Life Sciences and Medicine, Zhejiang Sci-Tech University, Hangzhou 310018, China

## Abstract

Genomic islands are related to microbial adaptation and carry different genomic characteristics from the host. Therefore, many methods have been proposed to detect genomic islands from the rest of the genome by evaluating its sequence composition. Many sequence features have been proposed, but many of them have not been applied to the identification of genomic islands. In this paper, we present a scheme to predict genomic islands using the chi-square test and random forest algorithm. We extract seven kinds of sequence features and select the important features with the chi-square test. All the selected features are then input into the random forest to predict the genome islands. Three experiments and comparison show that the proposed method achieves the best performance. This understanding can be useful to design more powerful method for the genomic island prediction.

## 1. Introduction

Horizontal gene transfer (HGT) is one of the main factors affecting bacterial adaptability. Hacker et al. found some viral gene clusters in *E. coli* genomes and did not exist in their close species, and they denoted them as pathogenic islands (PAIs) [1]. Since then, at least a dozen PAIs have been detected, such as “secretion island,” “antimicrobial island,” and “metabolic island” [2]. They are first expressed as genomic islands (GIs) and further encode them based on the functions related to the complex changes of niche [3]. For example, GIs are responsible for the type III secretion system, iron absorption function, toxin, and adhesion secretion, which enhance the survival ability of pathogens in the host body, leading to diseases [4, 5]. Some researchers reported that pathogenicity can be regulated by selective loss or recovery of specific GIs [6, 7], and PAI can be spontaneously removed from chromosomes at a detectable rate, resulting in different pathogenic phenotypes [8, 9]. Therefore, the detection of different GIs has become an important content of microbial evolution and function research.

With the help of large-scale comparative genomics, researchers found that GIs have different sequence composition, direct flanking duplication, mobility, and tRNA genes. In turn, exploring and utilizing these features can lead to better detection of GIs [3, 10–12]. GIs are scattered among close relatives, which carry some species patterns different from the host. Researchers can identify distant relatives by comparing the differences of 16S rRNA or other homologous sequences [13]. Some alignment-based methods have been developed to detect GIs, such as the basic local alignment method [14] and whole genome alignment method [15]. These tools rely on the observation that, compared with the conserved regions, the genomic regions that are not aligned across multiple genomes or only aligned with one genome are more likely to be hypothetical GIs. For some complex cases, several methods of constructing and applying multilayer or large-scale genome comparison are reported. For example, MobilomeFINDER first finds shared tRNA genes in several related genomes and then uses Mauve to search for GIs in the upstream and downstream regions of homologous tRNA genes [16]. Since the identified GIs with this method are related to tRNA disruption, the GIs without the tRNA gene as insertion site will be omitted. In order to solve this problem, MOSAIC has developed a method to identify strain-specific regions that do not necessarily insert tRNA [17]. Unfortunately, inversion and translocation are often mistaken for strain-specific regions. IslandPick is one of the most widely used tools for GI detection [18]. Given a genome, IslandPick first automatically selects the appropriate comparative genomes without any deviation and then uses Mauve to construct the whole genome alignment. To avoid duplication, IslandPick uses BLAST as a secondary filter to recheck the areas aligned by mauve. IslandPick has been integrated into the islandviewer website, where the dataset of precomputed GIs can be downloaded [19–21].

In addition to comparative genomics, component-based methods are also very sensitive to GI detection. Considering that GIs usually show significantly different sequence composition from the host, an effective detection algorithm can distinguish the abnormal region from the rest of the genome according to the composition deviation. In practice, component-based methods are desirable because they can rapidly detect GIs from analyzed sequences without the need for additional genomes. CG content and oligonucleotides with lengths 2-9 are widely used to describe the sequence composition in GI detection [10, 22–25]. For example, PAI-Finder calculates G + C content abnormality and codon usage deviation to detect GIs and further evaluates the candidate PAI only when PAI-like region partially or completely crosses GIs [26]. PAI Finder has been integrated into the PAI database, where comprehensive information of all annotated PAIs and predicted PAI in prokaryotic genome can be downloaded [27, 28]. The HMM model has also been introduced to detect abnormal areas containing component deviations [22, 29–31]. For example, SIGI-HMM constructs an HMM model to remove codons using biased ribosomal regions [29, 30], and IslandPath-DIMoB [31] uses HMM to identify migration genes by searching the PFAM37 migration gene map [32] of each prediction gene [11]. Alien_Hunter introduced a scoring system based on the *k*-mers and refined the boundary of prediction GIs using the HMM model [22].

Although the performance of the above algorithms is good, there are still some problems: (1) the comparative genomics relies heavily on the genomes used in the comparison, and so it can be used in the annotation process or when closely related genomes are available. Even if more genomes are available, researchers have to spend more time on selecting genomes from the species of interest. (2) Although these methods based on HMM show better performance in GI detection, they involve relatively more parameters and a lot of training calculation; so, it takes a long time to detect GIs. (3) In recent years, different sequence features have been proposed, but these features are rarely applied to genome island prediction. How to fuse and select some effective features is also a way to improve the efficiency of genomic island detection.

With the above problems in mind, we present a scheme to predict the genomic islands using the chi-square test and random forest algorithm. We first extract seven kinds of widely used sequence features and compare their performance in GI detection. The chi-square test is then used to select the important features. At last, all the selected features are input into the random forest to detect the genome islands. Through a comprehensive comparison and discussion, some novel valuable guidelines for use of the sequence features, feature selection, and prediction methods are obtained.

## 2. Materials and Methods

### 2.1. Datasets

Four standard data sets are used in this study. The first data set, PICK108, consists of 108 complete bacterial genome sequences and their annotations. The number of positive and negative GIs in this dataset is 3868 and 679, respectively [33]. The second set of data is referenced as CF15 which consists of 15 complete bacterial genome sequences and their annotations. The number of positive and negative GIs in this data set is 6070 and 5833, respectively [34]. The third data set, denoted as RGP104, consists of 104 complete bacterial genomes and their annotations. The number of positive and negative GIs is 1846 and 3267, respectively, in this dataset [35].

### 2.2. Sequence Features

Seven kinds of widely used sequence features are extracted for genome island detection. They are composition of *k*-spaced nucleic acid pairs (CKSNAP), dinucleotide composition (DNC), nucleic acid composition (NAC), pseudodinucleotide composition (PseDNC), electron-ion-interaction pseudopotentials of trinucleotide (PSEIIP), reverse compliment *k*-mer (RCKmer), and trinucleotide composition (TNC). The above features are obtained by iLearn that is a comprehensive python-based toolkit that integrates entity extraction, computation, entity analysis, and construction of predictor variables [36].

#### 2.2.1. Reverse Compliment *k*-Mer (RCKmer)

Reverse compliment *k*-mer is a variant of *k*-mer, which ignores the complementary sequences of adjacent nucleotide sequences. For example, there are 16 types of 2-mer: “AA,” “CC,” “GG,” “TT,”“AC,” “CA,” “GA,” “TA,” “AG,” “CG,” “GC,” “GT,” “AT,” “CT,” “TC,” and “TG.”. Because “TT” is the reverse completion *k*-mer of “AA,” it can be left out. Therefore, there are only 10 kinds of 2-mer in this method: “AA,” “CC,” “AC,” “CA,” “GA,” “AG,” “CG,” “GC,” “AT,” and “TA.” The frequency of each *k*-mer is calculated in turn [37].

#### 2.2.2. Composition of *k*-Spaced Nucleic Acid Pairs (CKSNAP)

CKSNAP feature represents the composition of nucleotide pairs that are separated by *k* (*k* =0, 1, 2, 5) nucleotides, and it reflects the short-range interactions of nucleic acids within the sequence [38]. Using *k* = 0 as an example, 16 0-spaced nucleotide pairs (i.e., “AA,” “AC,” “AG,” “AT,” “CA,” “CC,” “CT,” “CG,” “GA,” “GC,” “GG,” “GT,” “TA,” “TC,” “TG,” and “TT”) are generated. Then, a feature vector is defined as

(1) $$\begin{array}{c} {\left(\frac{N_{\text{AA}}}{N_{\text{Total}}},\frac{N_{\text{AC}}}{N_{\text{Tota}l}},\frac{N_{\text{AG}}}{N_{\text{Total}}},\frac{N_{\text{AT}}}{N_{\text{Total}}\;},\cdots ,\frac{N_{\text{TT}}}{N_{\text{Total}}}\right)}_{K=0}. \end{array}$$

In this study, all nucleotide pairs for *k* (0, 1,…, 5) were considered, and they are encoded to a 96-dimensional digital vector as follows:

(2) $$\begin{array}{c} {\left(\frac{N_{\text{AA}}}{N_{\text{Total}}},\frac{N_{\text{AC}}}{N_{\text{Total}}},\frac{N_{\text{AG}}}{N_{\text{Total}}},\cdots ,\frac{N_{\text{TT}}}{N_{\text{Total}}}\right)}_{K=0},\cdots ,{\left(\frac{N_{\text{AA}}}{N_{\text{Total}}},\frac{N_{\text{AC}}}{N_{\text{Total}}},\frac{N_{\text{AG}}}{N_{\text{Total}}},,\cdots ,\frac{N_{\text{TT}}}{N_{\text{Total}}}\right)}_{K=5}. \end{array}$$

#### 2.2.3. Dinucleotide Composition (DNC)

DNC expresses the composition of consecutive pairs of nucleotides [36, 39]. The coding of the DNC characteristics uses 16 descriptors defined as follows:

(3) $$\begin{array}{c} D\left(i,j\right)=\frac{N_{\left(ij\right)}}{N-1},i,j\in \left\{A,C,G,T\right\}, \end{array}$$

where *N*_*ij*_ donates the number of dinucleotides represented by nucleotide types *i* and *j*.

#### 2.2.4. Trinucleotide Composition (TNC)

TNC refers to the composition of three consecutive nucleotides in biological sequences [40]. The coding of TNC 64 descriptors described as follows: (“AAA,” “AAC,” “AAG,” “AAT,” …, “TTT”), which can be defined as

(4) $$\begin{array}{c} D\left(i,j,k\right)=\frac{N_{\left(ijk\right)}}{N-2},i,j,k\in \left\{A,C,G,T\right\}, \end{array}$$

where *N*_*ijk*_ donates the number of trinucleotide pairs represented by nucleotide types *i*, *j*, and *k*.

#### 2.2.5. Pseudodinucleotide Composition (PseDNC)

PseDNC converts the local sequence arrangement and global sequence information into the feature vector [39]. The PseDNC is expressed as follows:

(5) $$\begin{array}{c} P={\left(p_{1},p_{2},\cdots ,p_{16},p_{16+1},\cdots ,p_{16+\lambda }\right)}^{T},\\ p_{k}=\left\{\begin{array}{c} \frac{f_{k}}{\sum_{i=1}^{16}f_{i}+w\sum_{j=1}^{\lambda }\theta_{j}},\;\left(1\leq k\leq 16\right)\\ \frac{w\theta_{k-16}}{\sum_{i=1}^{16}f_{i}+w\sum_{j=1}^{\lambda }\theta_{j}},\;\left(17\leq k\leq 16+\lambda \right) \end{array},\right. \end{array}$$

where *f*_*k*_ (*k* = 1, 2 ⋯ 16) reflects the normalized frequency of occurrence of dinucleotides, *λ* represents the highest counted rank of the correlation along the biological sequences, *w* (0 to 1) is the weight factor, and *θ*_*j*_ (*j* = 1, 2 ⋯ *λ*) is the *j*-tier correlation factor, which is defined as

(6) $$\begin{array}{c} \theta_{1}=\frac{1}{L-2}\overset{L-2}{\underset{i=1}{\sum }}\Theta \left(R_{i}R_{i+1},R_{i+1}R_{i+2}\right),\\ \theta_{\lambda }=\frac{1}{L-1-\lambda }\overset{L-1-\lambda }{\underset{i=1}{\sum }}\Theta \left(R_{i}R_{i+1},R_{i+\lambda }R_{i+\lambda +1}\right), \end{array}$$

where the correlation function is defined as

(7) $$\begin{array}{c} \Theta \left(R_{i}R_{i+1},R_{j}R_{j+1}\;\right)=\frac{1}{u}\overset{u}{\underset{u=1}{\sum }}{\left(C_{u}\left(R_{i}R_{i+1}\right)-C_{u}\left(R_{j}R_{j+1}\right)\right)}^{2}, \end{array}$$

where *μ* denotes the number of physicochemical indexes, *C*_*u*_(*R*_*i*_*R*_*i*+1_) is the numerical value of the *u*^th^ physicochemical index of the dinucleotide *R*_*i*_*R*_*i*+1_, and *C*_*u*_(*R*_*j*_*R*_*j*+1_) denotes the corresponding value of the dinucleotide *R*_*j*_*R*_*j*+1_ at position *j*.

#### 2.2.6. Nucleic Acid Composition (NAC)

NAC assesses the frequency of each nucleic acid along the sequence. The frequencies of all 4 natural nucleic acids (i.e., “ACGT”) can be calculated:

(8) $$\begin{array}{c} f\left(t\right)=\frac{N\left(t\right)}{N}\;t\in \left\{A,C,G,T\right\}, \end{array}$$

where *N*(*t*) represents the number of nucleic acid type *t*, while *N* is the length of a nucleotide sequence [36].

#### 2.2.7. Electron-Ion-Interaction Pseudopotentials of Trinucleotide (PSeEIIP)

EIIPA, EIIPT, EIIPG, and EIIPC represent the EIIP measurements of nucleotides *A*, *T*, *G*, and *C*, respectively. The average EIIP of the trinucleotides in each sample is exploited for the construction of the feature vector, which is described as follows:

(9) $$\begin{array}{c} Q=\left[{\text{EIIP}}_{\text{AAA}}\times {\text{f}}_{\text{AAA}},{\text{EIIP}}_{\text{AAc}}\times {\text{f}}_{\text{AAc}},{\text{EIIP}}_{\text{AAG}}\times {\text{f}}_{\text{AAG}},{\text{EIIP}}_{\text{AAT}}\times {\text{f}}_{\text{AAT}}\right], \end{array}$$

where *f*_*xyz*_ represents the normalized frequency of the *i*^th^ trinucleotide, EIIIP_*xyz*_ = EIIP_*x*_ + EIIP_*y*_ + EIIP_*z*_ represents the EIIP value of a trinucleotide and *x*, *y*, *z* ∈ {*A*, *C*, *G*, *T*} [36].

### 2.3. Chi-Square Test

All kinds of sequence features will be fused together in order to improve the prediction efficiency, but the redundancy of different features cannot be ignored. Therefore, one of the primary tasks involved in genomic island prediction is to select the best features from the given dataset to achieve the best prediction. This work uses the chi-square test to select the best features for genomic island prediction.

The chi-square (*𝒳*^2^) test measures the deviation from the expected distribution [40, 41]. Statistically, *𝒳*^2^ tests the independence of two variables, where two variables *A* and *B* are defined as independent if *P*(*AB*) = *P*(*A*)*P*(*B*) or *P*(*A* | *B*) = *P*(*A*) (*P*(*B* | *A*) = *P*(*B*)). In feature selection, the two variables are the term occurrence and the class occurrence. The terms in relation to the quantity are classified as follows:

(10) $$\begin{array}{c} X^{2}\left(D,i,j\right)=\underset{w_{i}\in \left\{0,1\right\}}{\sum }\underset{w_{j}\in \left\{0,1\right\}}{\sum }\frac{{\left(N_{w_{i}w_{j}}-F_{w_{i}w_{j}}\right)}^{2}}{F_{w_{i}w_{j}}}, \end{array}$$

where *N* is the observed frequency in *D* and *F*. *w*_*i*_ and *w*_*j*_ are defined as

(11) $$\begin{array}{c} I\left(U,C\right)=\underset{w_{i}\in \left\{1.0\right\}}{\sum }\underset{w_{j}\in \left\{1.0\right\}}{\sum }P\left(U=w_{i},C=w_{j}\right)\log_{2}\frac{P\left(U={\text{w}}_{i},C=w_{j}\right)}{P\left(U=w_{i}\right)P\left(C=w_{j}\right)}, \end{array}$$

where *U* is a random variable that takes values *w*_*i*_ = 1 (the presence of the feature *i*) and *w*_*i*_ = 0 (absence of the feature *i*), and *C* is a random variable that takes values *e*_*j*_ = 1 (the presence of the feature in class *j*) and *e*_*j*_ = 0 (absence of the feature in class *j*). We write *U*_*i*_ and *U*_*j*_ if it is not clear from context which features *i* and class *j* we are referring to and got the following equation:

(12) $$\begin{array}{c} I\left(U,C\right)=\frac{F_{11}}{F}\log_{2}\frac{FF_{11}}{F_{1}F_{1}}+\frac{F_{01}}{F}\log_{2}\frac{{FF}_{01}}{F_{0}F_{1}}+\frac{F_{10}}{F}\log_{2}\frac{FF_{10}}{F_{1}F_{0}}+\frac{F_{00}}{F}\log_{2}\frac{{FF}_{00}}{F_{0}F_{0}}, \end{array}$$

where the *N* are counts of features that have the values of *w*_*i*_ and *w*_*j*_ that are indicated by the two subscripts. For example, *F*_10_ is the number of features that contain *i* (*w*_*i*_ = 1) and are not in *j*(*w*_*j*_ = 0). *F*_1_ = *F*_10_ + *F*_11_ is the number of features that contain *i* (*w*_*i*_ = 1), and we count features independent of class membership *w*_*i*_ ∈ {0, 1} . *F* = *F*_00_ + *F*_01_ + *F*_10_ + *F*_11_ is the total number of documents [42].

*𝒳* ^2^ is a measure of how much expected counts *E* and observed counts *N* deviate from each other. A high value of *𝒳*^2^ indicates that the hypothesis of independence, which implies that expected and observed counts are similar, is incorrect. An arithmetically simpler way of computing *𝒳*^2^ is the following:

(13) $$\begin{array}{c} X^{2}\left(D,i,j\right)=\frac{\left(F_{11}+F_{10}+F_{01}+F_{00}\right)*{\left(F_{11}+F_{00}-F_{10}F_{01}\right)}^{2}}{\left(F_{11}+F_{01}\right)*\left(F_{11}+N_{10}\right)*\left(F_{10}+F_{00}\right)*\left(F_{01}+F_{00}\right)}. \end{array}$$

### 2.4. Prediction Algorithm

Random forest (RF) is among the best classification algorithms and widely applied to manage many biological problems. It works by building small groups of weak classifiers, to finally combine them and form a strong classifier. This is a configuration learning method that can build models that create multiple decision trees during training and will remove modal classes from classes predicted by a single tree. It is a fusion of tree predictors, where each tree depends on the value of an independent sampled random vector and the same distribution of all trees in the forest [43].

A random forest is a collection of tree predictor *h*(*X*; *ω*_*i*_), *i* = 1, ⋯, *I*, where *X* represents the observed input (covariate) vector of length *p* with associated random vector *X*  and *ω*_*i*_. They are independent and identically distributed (*iid*) random vectors. As mentioned, we focus on the regression setting for which we have a numerical outcome *Y*, but we make some points of contact with classification (categorical outcome) problems [44]. The observed (training) data is assumed to be independently drawn from the joint distribution of (*X*, *Y*) and comprises *n*(*p* + 1)-tuples *X*(*x*_1_, *y*_1_), ⋯, (*x*_*n*_, *y*_*n*_).

For regression, the random forest prediction is the weighted average over the collection

(14) $$\begin{array}{c} h\left(y\right)=\left(\frac{1}{k}\right)\overset{I}{\underset{i=1}{\sum }}h\left(X;\omega_{i}\right). \end{array}$$

As *i* → ∞, the law of large numbers ensures

(15) $$\begin{array}{c} E_{X,Y}\left(Y-\bar{h}{\left(X\right)}^{2}\right.\to E_{X,Y}{\left(Y-E_{\omega }\bar{h}\left(X,\omega \right)\right)}^{2}. \end{array}$$

The quantity on the right is the prediction (or generalization) error for the random forest, denoted as *PE*_*f*_^∗^. The convergence implies that random forests do not overfit. Now, define the average prediction error for an individual tree *h*(*X*, *ω*)

(16) $$\begin{array}{c} {PE}_{t}^{*}=E_{\omega }E_{X,Y}{\left(Y-h\left(X,\omega \right)\right)}^{2}. \end{array}$$

Assume that for all the tree is unbiased, i.e., *EY* = *E*_*X*_*h*(*X*, *ω*). Then,

(17) $$\begin{array}{c} {PE}_{f}^{*}\leq \bar{\mu }PE_{t}^{*}, \end{array}$$

where $\bar{\mu }$ is the weighted correlation between residuals *Y* − *h*(*X*, *ω*) and *h*(*X*; *ω*) for independent *ω*, *ω*^*k*^. The above inequality pinpoints what is required for accurate random forest regression: low correlation between residuals of differing tree members of the forest and low prediction error for the individual trees [44]. Further, the random forest will decrease the individual tree error (*PE*_*t*_^∗^), by the factor $\bar{\mu }$.

### 2.5. Performance Evaluation

This work introduces crossvalidation to evaluate the proposed method and calculates accuracy, recall, *F*-measure, precision specificity, sensitivity, and precision as standard performance indicators. They are defined as follows:

(18) $$\begin{array}{c} \text{Acc}=\frac{\text{TP}+\text{TN}}{\text{TP}+\text{TN}+\text{FP}+\text{FN}}\times 100,\\ \text{Recall}=\frac{\text{TP}}{\text{TP}+\text{FN}}\times 100,\\ \text{Prec}=\frac{\text{TP}}{\text{TP}+\text{FP}},\\ \text{Sn}=\frac{\text{TP}}{\text{TP}+\text{FN}},\\ \text{Sp}=\frac{\text{TN}}{\text{TN}+\text{FP}},\\ F1=\frac{2\text{TP}}{2\text{TP}+\text{FP}+\text{FN}},\\ \text{MCC}=\frac{\text{TP}\times \text{TN}-\text{FP}\times \text{FN}}{\sqrt{\left(\text{TP}+\text{FP}\right)\left(\text{TP}+\text{FN}\right)\left(\text{TN}+\text{FP}\right)\left(\text{TN}+\text{FN}\right)}}, \end{array}$$

where TP is the number of true positives, FP is the number of false positives, TN is the number of true negatives, and FN is the number of false negatives.

## 3. Results and Discussion

### 3.1. Performance of the Proposed Prediction Method

**Figure 1:**
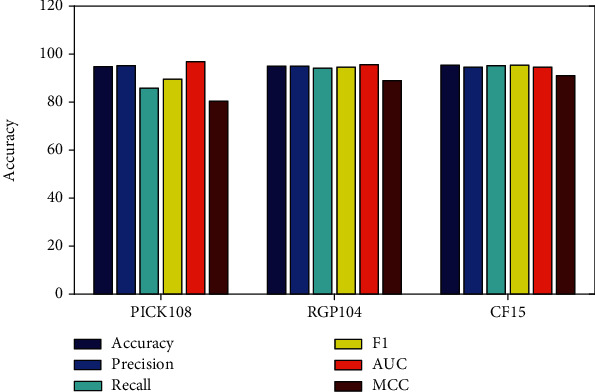
Comparison of the accuracy, precision, recall, F1, AUC, and MCC of the PICK108, CF15, and RGP104 datasets.

To build the prediction model, seven kinds of sequence features are extracted, fused, and filtered by the chi-square test and then input into the random decision tree for genomic island prediction. Accuracy, F1, MCC, precision, recall, and AUC are calculated based on 10 times crossvalidation, which are summarized in Figure 1.

Figure 1 shows that the proposed method achieves good performance among four datasets. As for PICK108, its accuracy, precision, recall, F1, AUC, and MCC are 94.6%, 95.1%, 85.7%, 89.5%, 96.8%, and 80.3%, respectively. For dataset CF15, the overall precision is 94.9%, and precision, recall, F1, AUC, and MCC are 94.8%, 94.0%, 94.4%, 95.6%, and 88.8%, respectively. As for RGP104, its accuracy, precision, recall, F1, AUC, and MCC are 95.4%, 94.4%, 95.2%, 95.4%, 94.5%, and 90.9%, respectively.

We further compare the proposed method with the current methods. For the convenience of comparison, we compare our results with that of the published results with the existing methods. Therefore, different datasets choose different evaluation methods, which are summarized in Tables 1–3.

As for PICK108, the proposed method is compared with the Centroid [45], INDeGenIUS [46], MTGIpick [33], SigHunt [47], and Zisland Explore [48].  Table 1 indicates that the proposed method achieves the highest accuracy, precision, and recall with the values of 94.6%, 95.1%, and 85.7%, respectively. Compared with the second best method, the accuracy, precision, and recall of the proposed method are 8.4%, 22.3%, and 38.5% higher than that of MTGIpick, respectively.

In the RGP104 dataset, PanRGP [35], IslandViewer [19, 20], IslandPath-Dimob [31], IslandCafe, and SIGI-HMM [29, 30] are compared with the proposed method.  Table 2 shows that the proposed method outperforms the others in term of MCC, F1, accuracy, and recall. Specifically, the MCC, F1, ACC, and recall of the proposed method are 11%, 12.4%, 3.2%, and 15.2%, respectively, higher than that of the PanRGP model [35], but its accuracy is 0.1% lower than that of the PanRGP model.

In the CF15 experiment, IslandCafe [34], IslandViewer [19, 20], IslandPath-Dimob [31], Zisland Explorer [48] and SIGI-HMM [29, 30] are compared with the proposed method.  Table 3 indicates that the proposed method achieves the highest recall, precision, F1, and MCC with the values of 95.4%, 95.4%, 95.4%, and 90.9%, respectively, which are 23.4%, 28.4%, 29.4%, and 28.9% higher than that of the next competitive method [34].

The above results show that the proposed method outperforms the available genomic island prediction methods, indicating that the combination of different features, feature selection based on the chi-square test, and prediction algorithm is very effective to advance the prediction. This understanding can be used to develop more powerful genomic island prediction methods.

### 3.2. Influence of the Different Features

**Figure 2:**
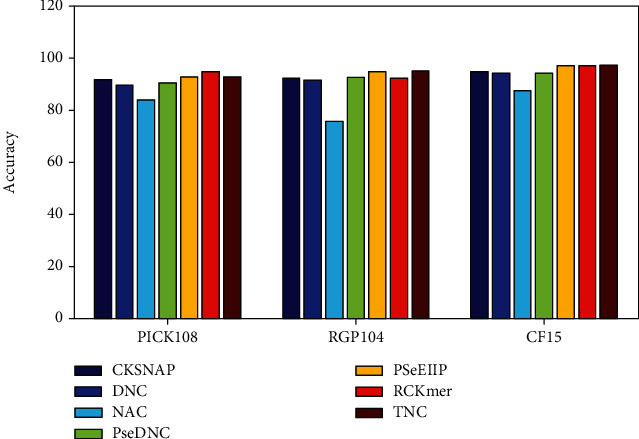
Comparison of the overall prediction accuracies of seven kinds of the sequence features.

To predict genomic islands, we use seven kinds of protein features: reverse compliment *k*-mer (RCKmer), composition of *k*-spaced nucleic acid pairs (CKSNAP), dinucleotide composition (DNC), trinucleotide composition (TNC), pseudodinucleotide composition (PseDNC), nucleic acid composition (NAC), and electron-ion-interaction pseudopotentials of trinucleotide (PSeEIIP). To evaluate the contribution of each kind of the sequence features, we present the comparison of the accuracies of seven kinds of the sequence features in Figure 2.

Figure 2 indicates that each feature makes its own positive contributions to the predictions; although, different features have certain preferences for different data sets. On the whole, PSeEIIP, RCKmer, and TNC achieve the best performance among all kinds of the sequence features. It is easy to note that PSeEIIP and RCKmer not only reflect the content of components but also focus the local sequence arrangement and global sequence information and calculate the energy of delocalized electrons in nucleotides as the electron-ion interaction. Compared with the ANC and DNC, PSeEIIP and RCKmer are more closely related to the genomic islands, and this is why they achieve the better performance in the genomic island prediction.

### 3.3. Influence of the Different Feature Selections

**Figure 3:**
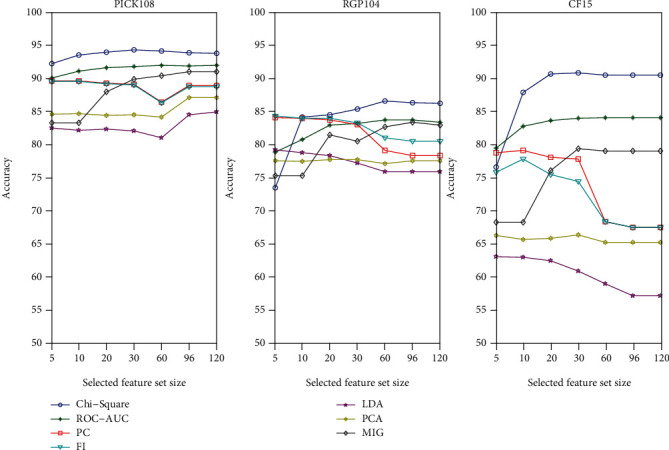
The comparison of the overall accuracies of all experiments with the selected feature sets for three datasets.

A feature of the proposed method is the feature selection based on the chi-square test. For a better understanding of the feature selection, we select the feature set with size from 5 to 120. All experiments are performed with each selected feature set using the 10 times crossvalidation test, and overall accuracy is chosen to represent the score in this prediction.  Figure 3 is the overall accuracies of all experiments with the selected feature sets for three datasets.

As would be expected, the overall accuracy first increases and then decreases as the selected feature size continues to increase. When the selected feature set size is less than 30, all data sets have reached the best prediction. As the increase of the number of selected features, the overall accuracy decreases. The chi-square is further compared with feature importance (FI), Pearson correlation (PC), ROC-AUC, mutual information gain (MIG), linear discriminant analysis (LDA), and principal component analysis (PCA), and it is easy to note that the chi-square test achieves the best performance among seven feature selection method.

### 3.4. Influence of the Different Prediction Algorithms

**Figure 4:**
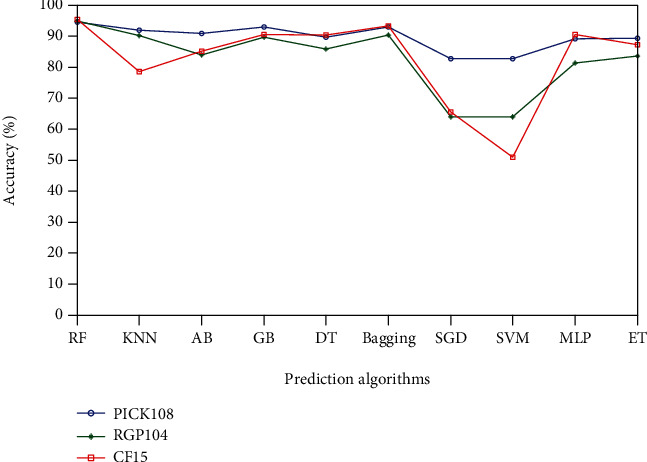
The comparison of the overall accuracies of different prediction algorithms with the selected feature sets for three datasets.

Random forest (RF) was employed as a classifier in this work. To compare different classifiers' performance, support vector machine (SVM), *k*-nearest neighbor (KNN), gradient boosting (GB), adaBoost (AB), decision tree (DT), bagging, extra trees (ET), stochastic gradient descent (SGD), and layer perceptron (MLP) were also adopted for protein structural class prediction. All experiments are performed with each selected feature set using the 10 times crossvalidation test, and overall accuracy is chosen to represent the score in this prediction.  Figure 4 summarizes the overall accuracies of all experiments with the different prediction algorithms for three datasets.

From Figure 4, it is easy to note that the random forest (RF) achieves the best performance among the ten classifiers. Specifically, the average overall prediction accuracy is 95% for PICK108, RGP104, and CF15 datasets compared with 91% of the gradient boosting (GB) and 92% of the bagging. These results indicate that the random forest is a more powerful classifier for the genomic island prediction.

## 4. Conclusion

Genome islands are related to the rapid adaptation of prokaryotes, which have important medical, economic, or environmental significance. Some methods usually evaluate all features and focus on whether the local features of a certain area are significantly different from the host. Although these methods have achieved good experimental results, various feature extraction methods have been proposed, but they are rarely used to predict genomic islands. With these problems in mind, we present a scheme to predict the genomic islands using the chi-square test and random forest algorithm. We extract seven kinds of widely used sequence features and select the important features with the chi-square test. At last, all the selected features are input into the random forest to predict the genome islands. Three experiment results show that the proposed method has better performance than previous methods.

The first contribution can be seen from the influence of the different features, and we find that PSeEIIP, RCKmer, and TNC are more closely related to the genomic islands and achieve the best performance among all kinds of the sequence features. The second contribution can be indicated from the influence of the different feature selections, and the chi-square test achieves the best performance among seven feature selection method. The final contribution can be seen from the influence of the different prediction algorithms, and we notice that the random forest (RF) achieved the best performance among the ten classifiers; its accuracy is 3% higher than that of the next one. This understanding can be then used to develop more powerful methods for genomic island prediction.

## Figures and Tables

**Table 1:** Comparison of the proposed method with other reported results on the PICK108 dataset.

Method	Accuracy	Precision	Recall
Centroid	82.4	61.4	27.6
INDeGenIUS	82.4	67.9	19.9
MTGIpick	86.2	72.8	47.2
SigHunt	80.5	51.0	24.0
Zisland Explorer	83.8	75.9	25.5
This paper	94.6	95.1	85.7

**Table 2:** Comparison of the proposed method with other reported results on the RGP104 dataset.

Method	MCC	F1	ACC	Precision	Recall
PanRGP	77.8	80.9	92.4	94.9	76.4
IslandViewer	76.2	82.0	91.1	90.8	78.8
IslandPath	52.3	57.0	78.1	89.1	47.7
IslandCafe	37.7	44.4	76.1	76.9	35.5
SIGI-HMM	33.8	45.5	75.6	65.5	37.6
This paper	88.8	94.4	95.6	94.8	94.0

**Table 3:** Comparison of the proposed method with other reported results on the CF15 dataset.

Method	Recall	Precision	F1	MCC
IslandCafe	71.0	61.0	66.0	62.0
IslandViewer	72.0	59.0	65.0	59.0
IslandPath-Dimob	53.0	67.0	59.0	55.0
Zisland Explorer	45.0	56.0	50.0	46.0
SIGI-HMM	24.0	57.0	33.0	32.0
This paper	95.4	95.4	95.4	90.9

## Data Availability

All the data used to support the findings of this study are available on https://github.com/Onesime243/Chi_square_Genomic_Islands_predicton_data-and-result.git.
